# Supplementary material for: Constructing Randomly Lamellar HKUST–1@Clinoptilolite through Polyethylene Glycol—Assisted Hydrothermal Method and Coordinated Complexation for Enhanced Adsorptive Separation for CO2 and CH4
Source: Nanomaterials (Basel). 2023 Jun 14;13(12):1860. doi: 10.3390/nano13121860 (PMC10303906; doi:10.3390/nano13121860)
Supplement: Supplementary file 1 [file nanomaterials-13-01860-s001.zip › nanomaterials-2311751-supplementary.pdf]

**Constructing randomly lamellar HKUST–1@clinoptilolite through polyethylene glycol–assisted hydrothermal method and coordinated complexation for enhanced adsorptive separation for $CO_2$ and $CH_4$**

Mingxuan Zhang, Jiawei Zhou, Chunlei Wan, Ming Liu, Xia Wu*, Jihong Sun*

Beijing Key Laboratory for Green Catalysis and Separation, Department of Chemical Engineering, Beijing University of Technology, Beijing, 100124, China

**Electronic Supplementary Information**

**Figure S1.** Schematic of the PEG-assisted synthesis of CP via crystallization and delamination process.

**Figure S2.** XRD patterns of (A): (a) pure CP, (b) CP-0, (c) CP-24, (d) CP-72, (e) CP-72-E, and (f) HKUST-1 and (B): (a) Cu-CP-0-E-10.5, (b) Cu-CP-24-E-10.1, (c) Cu-CP-72-E-3.4, and (d) Cu-CP-72-E-7.3.

**Figure S3.** SAXS patterns of (a) pure CP, (b) CP-0, (c) CP-24, (d) CP-72, (e) CP-72-E, (f) Cu-CP-72-E-3.4, and (g) Cu-CP-72-E-7.3.

**Figure S4.** The pair distance distribution function (P(r)~r) profiles of (A): (a) pure CP, (b) CP-0, (c) CP-24, (d) CP-72, and (e) CP-72-E, and (B): (a) Cu-CP-72-E-3.4, and (b) Cu-CP-72-E-7.3.

**Figure S5.** FT-IR spectra of (a) CP-0, (b) CP-24, (c) CP-72, (d) CP-72-E, (e) Cu-CP-72-E-7.3, (f) HKUST-1@CP-72-E-7.3, and (g) HKUST-1.

**Figure S6.** $N_2$ adsorption-desorption isotherms of (A): (a) pure CP, (b) CP-0, (c) CP-24, (d) CP-72, (e) CP-72-E, (f) Cu-CP-72–E-3.4, and (g) Cu-CP-72-E-7.3 and (B): (a) HKUST-1, (b) HKUST-1@CP-0-E-10.5, (c) HKUST-1@CP-24-E-10.1, (d) HKUST-1@CP-72-E-3.4, and (e) HKUST-1@CP-72-E-7.3.

**Figure S7.** Crystallization kinetic profiles of the pure CP (A) and CP-X (B) at different temperatures: (a) 140 °C, (b) 150 °C, and (c) 160 °C.

**Figure S8.** XRD patterns of A: (a) HKUST-1@CP-0-E-10.5, (b) HKUST-1@CP-24-E-10.1, (c) HKUST-1@CP-72-E-3.4, and (d) HKUST-1@CP-72-E-7.3. B: (a) Cu-CP-0-E-10.5, (b) Cu-CP-24-E-10.1, (c) Cu-CP-72-E-3.4 and (d) Cu-CP-72-E-7.3.

**Figure S9.** TG curves of (a) pure CP, (b) CP-72, (c) CP-72-E, (d) Cu-CP-72-E-7.3, (e) HKUST-1@CP-72-E-7.3, and (f) HKUST-1.

**Figure S10.** Equilibrium adsorbed isotherms of the samples using $CO_2$ as adsorbate at 273 K (A) and 298 K (B), respectively; $CH_4$ as adsorbate at 273 K (C) and 298 K (D), respectively: (a) pure CP, (b) CP-0, (c) CP-24, (d) CP-72, and (e) CP-72-E; $CO_2$ as adsorbate at 273 K (E) and 298 K (F), respectively; $CH_4$ as adsorbate at 273 K (G) and 298 K (H), respectively: (a) Cu-CP-72-E-3.4, and (b) Cu-CP-72-E-7.3.

**Table S1.** Summaries of prepared conditions and various parameters of the obtained Cu-CPs.

**Table S2.** Summaries of amounts of the used ligand and triethylamine.

**Table S3.** Summaries of the textural parameters of the related samples.

**Table S4.** Summaries of various kinetic parameters during CP crystallization.

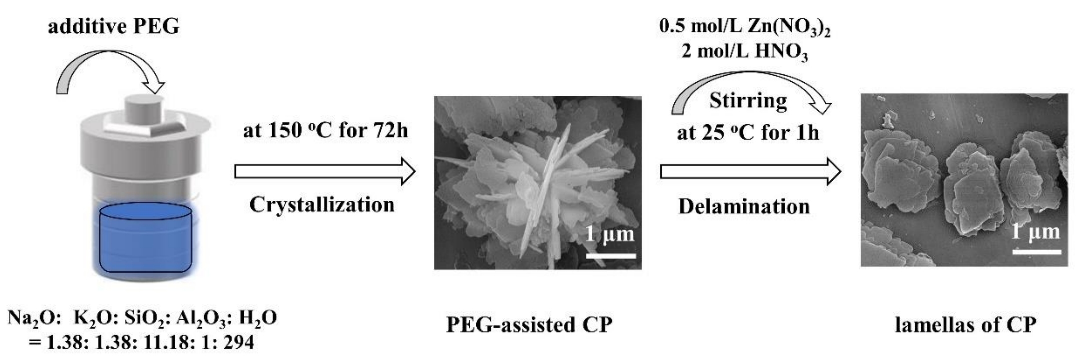


**Figure S1**. Schematic of the PEG-assisted synthesis of CP via crystallization and delamination process.

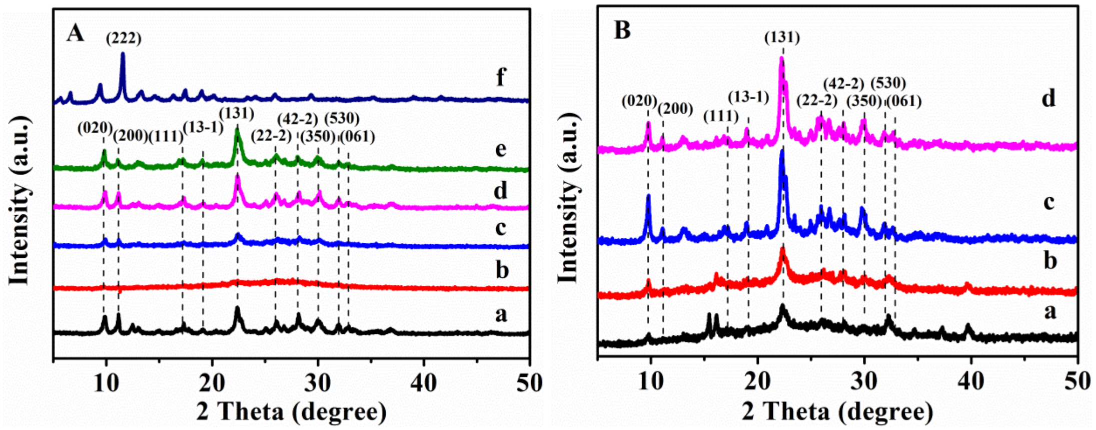


**Figure S2.** XRD patterns of (A): (a) pure CP, (b) CP-0, (c) CP-24, (d) CP-72, (e) CP-72-E, and (f) HKUST-1 and (B): (a) Cu-CP-0-E-10.5, (b) Cu-CP-24-E-10.1, (c) Cu-CP-72-E-3.4, and (d) Cu-CP-72-E-7.3.

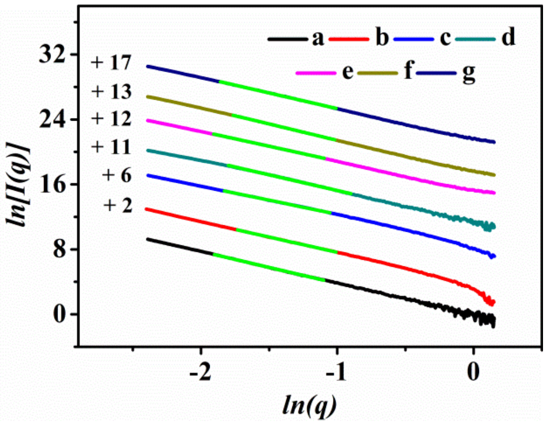


**Figure S3.** SAXS patterns of (a) pure CP, (b) CP-0, (c) CP-24, (d) CP-72, (e) CP-72-E, (f) Cu-CP-72-E-3.4, and (g) Cu-CP-72-E-7.3.

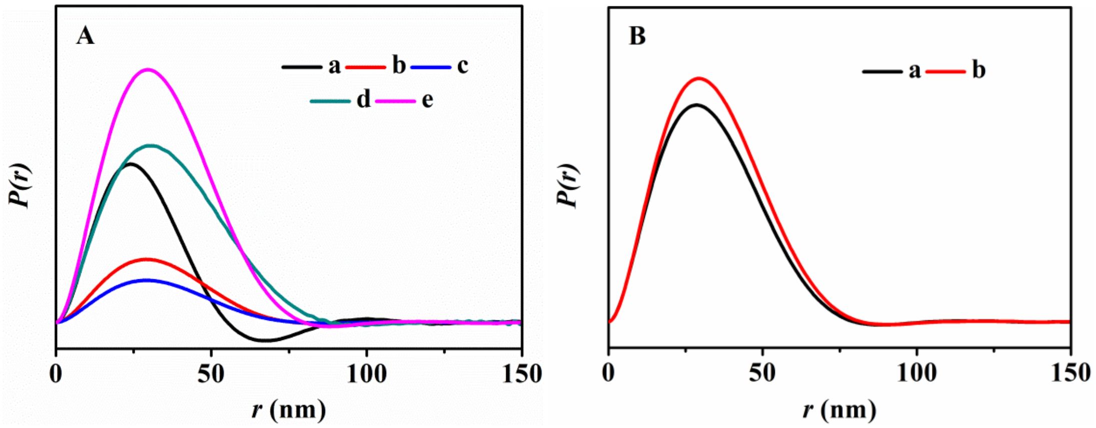


**Figure S4.** The pair distance distribution function (P(r)~r) profiles of (A): (a) pure CP, (b) CP-0, (c) CP-24, (d) CP-72, and (e) CP-72-E, and (B): (a) Cu-CP-72-E-3.4, and (b) Cu-CP-72-E-7.3.

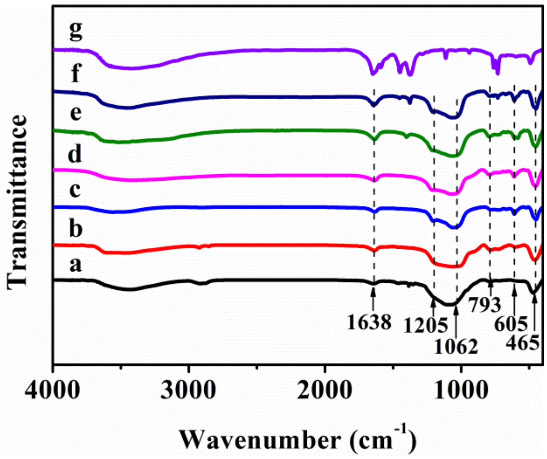


**Figure S5.** FT-IR spectra of (a) CP-0, (b) CP-24, (c) CP-72, (d) CP-72-E, (e) Cu-CP-72-E-7.3, (f) HKUST-1@CP-72-E-7.3, and (g) HKUST-1.

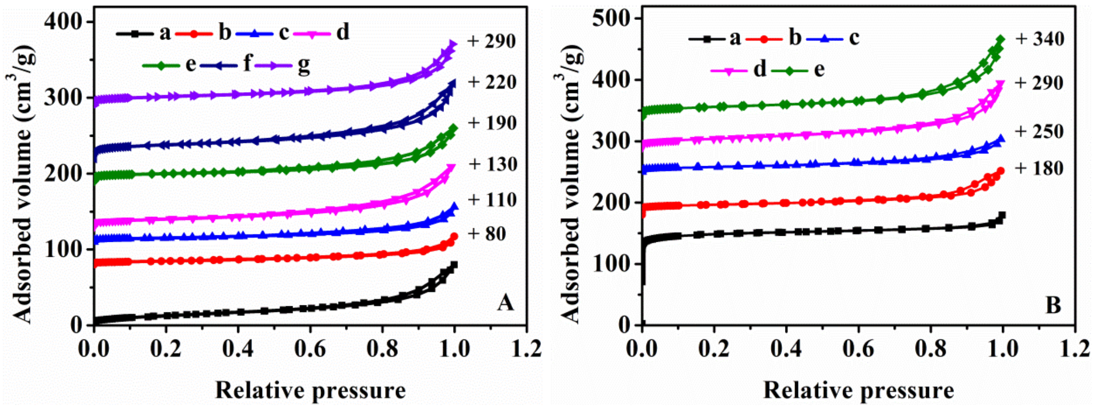


**Figure S6.** $N_2$ adsorption-desorption isotherms of (A): (a) pure CP, (b) CP-0, (c) CP-24, (d) CP-72, (e) CP-72-E, (f) Cu-CP-72–E-3.4, and (g) Cu-CP-72-E-7.3 and (B): (a) HKUST-1, (b) HKUST-1@CP-0-E-10.5, (c) HKUST-1@CP-24-E-10.1, (d) HKUST-1@CP-72-E-3.4, and (e) HKUST-1@CP-72-E-7.3.

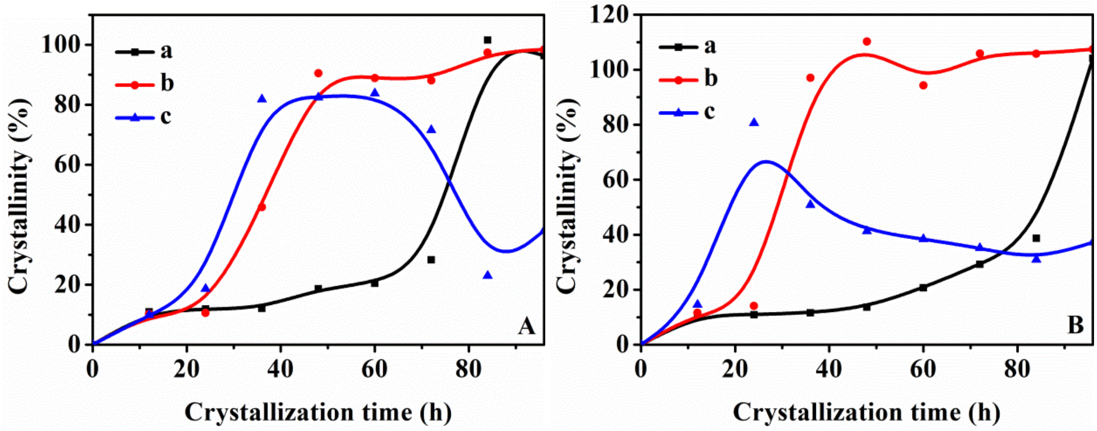


**Figure S7.** Crystallization kinetic profiles of the pure CP (A) and CP-X (B) at different temperatures: (a) 140 °C, (b) 150 °C, and (c) 160 °C.

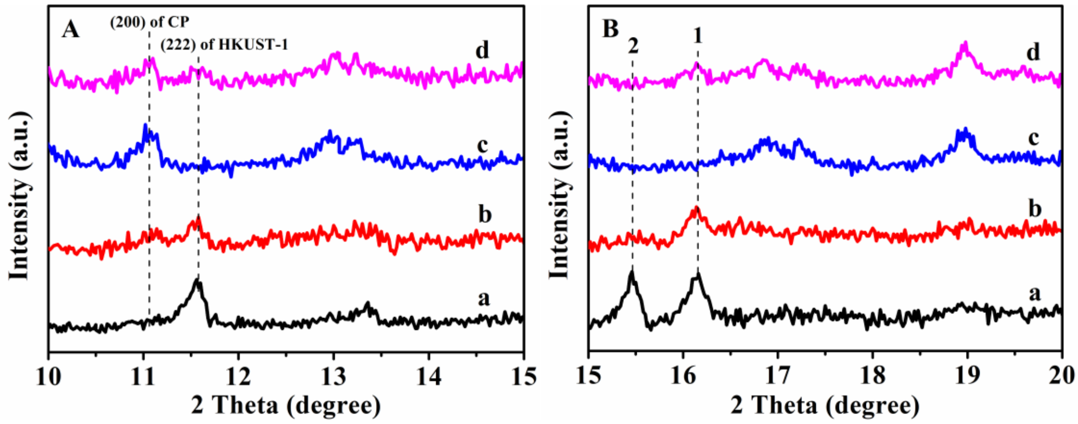


**Figure S8.** XRD patterns of A: (a) HKUST-1@CP-0-E-10.5, (b) HKUST-1@CP-24-E-10.1, (c) HKUST-1@CP-72-E-3.4, and (d) HKUST-1@CP-72-E-7.3. B: (a) Cu-CP-0-E-10.5, (b) Cu-CP-24-E-10.1, (c) Cu-CP-72-E-3.4 and (d) Cu-CP-72-E-7.3.

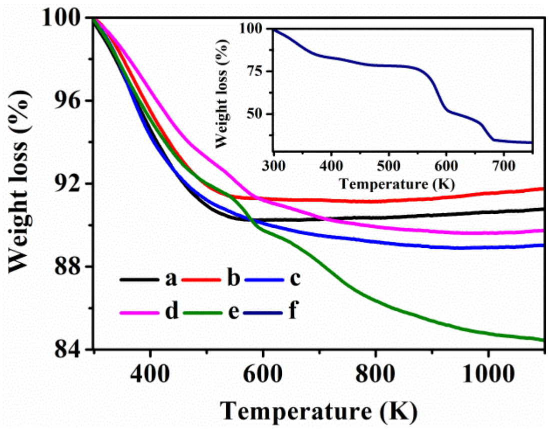


**Figure S9.** TG curves of (a) pure CP, (b) CP-72, (c) CP-72-E, (d) Cu-CP-72-E-7.3, (e) HKUST-1@CP-72-E-7.3, and (f) HKUST-1.

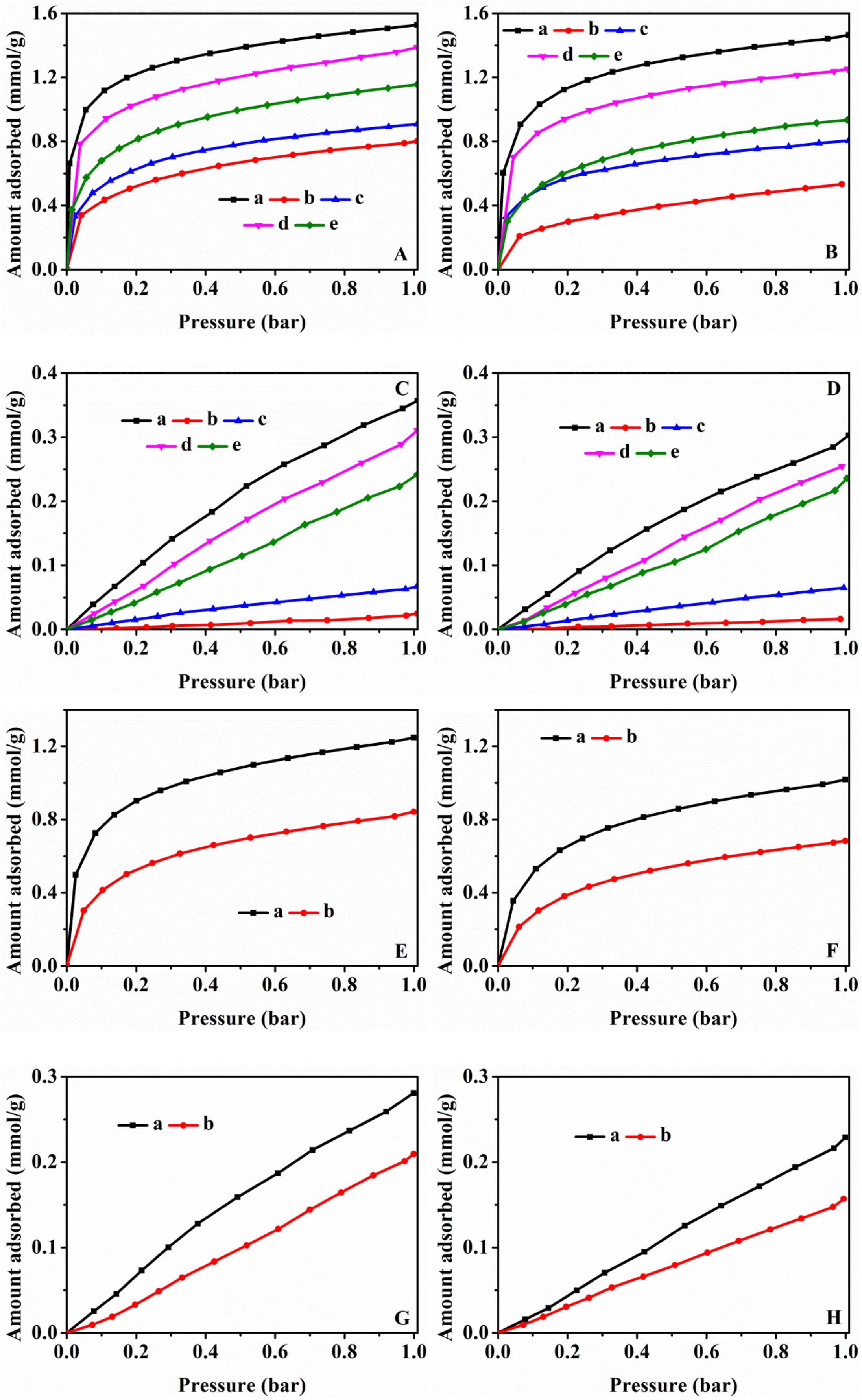
A
B
C
D
E
F
G
H
a
b
c
d
e
Amount adsorbed (mmol/g)
Pressure (bar)
0.0
0.2
0.4
0.6
0.8
1.0
1.2
1.6
0.1
0.3

**Figure S10.** Equilibrium adsorbed isotherms of the samples using $CO_2$ as adsorbate at 273 K (A) and 298 K (B), respectively; $CH_4$ as adsorbate at 273 K (C) and 298 K (D), respectively: (a) pure CP, (b) CP-0, (c) CP-24, (d) CP-72, and (e) CP-72-E; $CO_2$ as adsorbate at 273 K (E) and 298 K (F), respectively; $CH_4$ as adsorbate at 273 K (G) and 298 K (H), respectively: (a) Cu-CP-72-E-3.4, and (b) Cu-CP-72-E-7.3.

**Table S1.** Summaries of prepared conditions and various parameters of the obtained Cu-CPs.

| Sample | $c(CuCl_2)$ ($mol \cdot L^{-1}$) | Exchange times | (Na and K) Content ($mmol \cdot g^{-}1$) | Cu content (wt%) | Crystallinity (%) |
|---|---|---|---|---|---|
| Cu-CP-0-E-10.5 | 0.06 | 10 | 2.55 | 10.5 | 20.1 |
| Cu-CP-24-E-10.1 | 0.06 | 10 | 2.89 | 10.1 | 41.1 |
| Cu-CP-72-E-3.4 | 0.06 | 1 | 2.72 | 3.4 | 93.3 |
| Cu-CP-72-E-7.3 | 0.06 | 10 | 2.72 | 7.3 | 87.8 |

**Table S2.** Summaries of amounts of the used ligand and triethylamine.

| Sample | Cu quantity (g) | Ligand Amount (g) | Tritheylamine amont (mL) |
|---|---|---|---|
| Cu-CP-0-E-10.5 | 0.032 | 0.07 | 0.3 |
| Cu-CP-24-E-10.1 | 0.030 | 0.07 | 0.3 |
| Cu-CP-72-E-3.4 | 0.010 | 0.03 | 0.1 |
| Cu-CP-72-E-7.3 | 0.022 | 0.05 | 0.2 |

**Table S3.** Summaries of the textural parameters of the related samples.

| Sample | $S_{BET}$ [a] ($m^2/g$) | $S_{micro}$ [b] ($m^2/g$) | $V_{total}$ [c] ($cm^3/g$) | $d_p$ [d] (nm) |
|---|---|---|---|---|
| pure CP | 42.0 | 0.90 | 0.12 | 11.76 |
| CP-0 | 15.3 | 4.94 | 0.06 | 14.93 |
| CP-24 | 17.5 | 3.82 | 0.07 | 16.34 |
| CP-72 | 33.9 | 3.11 | 0.12 | 14.40 |
| CP-72-E | 35.9 | 11.09 | 0.11 | 12.03 |
| HKUST-1 | 516.1 | 27.44 | 0.28 | 1.94 |
| Cu-CP-72-E-3.4 | 63.8 | 21.57 | 0.15 | 9.58 |
| Cu-CP-72-E-7.3 | 39.5 | 8.39 | 0.13 | 12.63 |
| HKUST-1@CP-0-E-10.5 | 59.9 | 31.90 | 0.11 | 7.41 |
| HKUST-1@CP-24-E-10.1 | 27.3 | 0.73 | 0.08 | 12.13 |
| HKUST-1@CP-72-E-3.4 | 47.3 | 1.15 | 0.16 | 13.70 |
| HKUST-1@CP-72-E-7.3 | 55.2 | 13.50 | 0.20 | 14.16 |

[a] Multipoint BET surface area;

[b] Micropore surface area by t-plot;

[c] Total pore volume at $P/P_0 = 0.98$;

[d] Mean pore size obtained on the basis of BJH model using desorption branch of the isotherms.

**Table S4.** Summaries of various kinetic parameters during CP crystallization.

| Sample | Temperature (°C) | Induce stage | | | Growth period | | |
|---|---|---|---|---|---|---|---|
| | | $E_n$ (kJ/mol) | $lnA_n$ | $t_0$ (h) | $k_{max}$ | $E_g$ (kJ/mol) | $lnA_g$ |
| pure CP | 140 | 63.0 | 14.6 | 42 | 2.4 | 59.8 | 18.3 |
| | 150 | | | 27 | 3.7 | | |
| | 160 | | | 18 | 5.3 | | |
| CP−X | 140 | 106.2 | 27.0 | 50 | 5.4 | 31.3 | 10.8 |
| | 150 | | | 24 | 6.9 | | |
| | 160 | | | 12 | 8.3 | | |
